# Supplementary material for: Analyzing Molecular Determinants of Nanodrugs’ Cytotoxic Effects
Source: Int J Mol Sci. 2025 Jul 11;26(14):6687. doi: 10.3390/ijms26146687 (PMC12294593; doi:10.3390/ijms26146687)
Supplement: Supplementary file 1 [file ijms-26-06687-s001.zip › Cytotoxicity Checklist.pdf]

Supplementary Material. Checklist for minimizing nanodrug-induced toxicity. This checklist is intended for use during nanodrug design and development.

1. Targeting Strategy Selection

- ✓ Prioritize active targeting (e.g., receptor-mediated) over passive targeting (e.g., EPR effect) to improve tumor selectivity and reduce systemic toxicity.
- ✓ Functionalize nanocarriers with ligands such as: aptamers, folate, transferrin, PSMA (Prostate-Specific Membrane Antigen), etc., depending on target specificity.
- ✓ Evaluate receptor expression (e.g., HSP70, TfR1, integrins) in target tissue.

Passive targeting considerations:

- ✓ Assess EPR effect limitations and tumor heterogeneity;
- ✓ Consider vascular density variations in target tissues;
- ✓ Evaluate extracellular matrix composition impact;
- ✓ Account for interstitial pressure variations;
- ✓ Implement imaging assessments for personalized delivery;
- ✓ Design tailored nanocarriers for specific tumor microenvironments

2. Physicochemical Property Optimization

Size optimization

Avoid ultra-small particles: Stay above 1.4 nm (high toxicity threshold).

Target optimal range: > 15 nm diameter for reduced toxicity

Consider size impact on: surface area-to-volume ratio; cellular uptake efficiency; intracellular component interactions; immune system recognition.

Surface charge

Minimize positive surface charge to reduce: electrostatic interactions with cell membranes; membrane disruption potential; non-specific cellular uptake.

Consider neutral or slightly negative charges.

Validate charge effects on biodistribution.

Shape considerations

Prioritize spherical particles for: predictable macrophage internalization; reduced frustrated phagocytosis.

Avoid high-aspect-ratio structures (e.g., needle-like carbon nanotubes).

Minimize shape-induced persistent cellular stress.

Material selection

Prefer safer material classes: biodegradable polymers over persistent materials; protected quantum dots (CdSe/ZnS) over bare cores (CdTe); FDA-approved materials when possible.

Avoid or minimize: heavy metal components; cadmium-based quantum dots; highly hydrophobic surfaces.

3. Surface Modification Strategies

PEGylation implementation: apply PEGylation to prevent non-specific interactions; monitor for potential immunogenic reactions; assess biodistribution alterations; validate reduced cytotoxicity while maintaining efficacy.

Protein corona effect: characterize protein corona formation in relevant biological fluids; assess impact on cellular recognition, immune system activation, targeting specificity; design surface modifications to control corona composition.

Biodegradable coatings: implement biodegradable surface coatings; ensure controlled degradation profiles; validate biocompatibility of degradation products.

4. Cytotoxicity Mechanism Prevention

Oxidative stress mitigation

Design to minimize ROS generation: avoid surface redox-active materials; prevent photoactivation-induced ROS; minimize disruption of cellular antioxidant defenses.

Monitor key indicators: glutathione depletion; mitochondrial membrane potential loss; lipid peroxidation markers; DNA damage markers.

Consider protective strategies: antioxidant co-delivery; ROS scavenger incorporation.

Inflammatory response control

Minimize activation of inflammatory pathways: NF-κB signaling; MAPK pathways; PI3K/Akt pathways.

Prevent inflammasome activation: NLRP3 inflammasome; IL-1β release; pyroptotic cell death.

Monitor cytokine profiles: TNF-α levels; IL-6 and IL-8 release; Nitric oxide production; iNOS expression.

Consider anti-inflammatory strategies: NSAID co-delivery; pathway inhibitor incorporation.

Lysosomal dysfunction prevention

Minimize lysosomal accumulation: avoid cationic surface charges; control acid-sensitive moiety design; prevent lysosomal membrane permeabilization (LMP)

Maintain lysosomal integrity: preserve pH regulation; support autophagic flux; maintain exocytosis function.

Monitor lysosomal health: cathepsin leakage; membrane stability; enzymatic activity; autophagy markers.

5. Formulation-Specific Considerations

Liposomal formulations

Optimize lipid composition for reduced membrane disruption.

Control particle size distribution.

Implement surface modifications for targeting.

Monitor for lysosomal membrane interactions.

Polymer-drug conjugates

Select biodegradable polymer backbones.

Control molecular weight and branching.

Optimize drug loading and release kinetics.

Minimize lysosomal accumulation.

Inorganic nanoparticles

Metal NPs: focus on ROS prevention and oxidative stress control.

Quantum dots: ensure core protection and prevent heavy metal release.

Metal Oxides: control ROS generation and Fenton-type reactions.

Carbon nanomaterials

Minimize inflammatory potential.

Control aspect ratio and surface functionalization.

Address immune recognition and macrophage activation.

6. Safety Assessment and Risk Evaluation

Toxicity Index Scoring (0-21 point system)

Physicochemical properties assessment: particle size scoring; surface charge evaluation; shape factor analysis; material composition risk.

Biological response indicators: oxidative stress levels; inflammatory cytokine release; organelle damage markers.

Surface modification factors: PEGylation status; biodegradable coating presence; protein corona characteristics.

Risk categorization:

Low Risk (0-7 points): Proceed with standard safety protocols;

Moderate Risk (8-14 points): Implement enhanced safety measures;

High Risk (15-21 points): Redesign formulation or discontinue.

7. Validation and Testing Protocol

In vitro screening

Cell viability assays: IC_{50} determination across relevant cell lines.

Mechanistic studies: ROS generation measurement; inflammatory marker assessment; lysosomal integrity evaluation; mitochondrial function testing.

Comparative analysis: Free drug vs. nanodrug formulation.

Biomarker monitoring

Oxidative stress markers: glutathione levels; lipid peroxidation products; DNA damage indicators.

Inflammatory markers: cytokine profiles (TNF-α, IL-6, IL-8); NF-κB activation; MAPK pathway activity.

Cellular integrity markers: lysosomal enzyme leakage; mitochondrial membrane potential; cell death pathway activation.

Dose-response optimization

Establish maximum tolerated dose (MTD);

Determine therapeutic window;

Compare with conventional formulations;

Assess dose-dependent toxicity mechanisms.

8. Therapeutic Intervention Strategies

Protective co-treatments

ROS scavengers: N-acetylcysteine and other ROS scavengers, antioxidants.

Anti-inflammatory agents: NSAIDs, pathway inhibitors.

Cellular protective agents: Membrane stabilizers.

Formulation optimization

Implement combination approaches for synergistic protection.

Optimize release kinetics to minimize peak toxicity.

Consider sequential delivery strategies.

9. Documentation and Compliance

Safety documentation: complete physicochemical characterization; comprehensive toxicity profile; mechanism of action documentation; risk-benefit analysis; comparative safety assessment.

Regulatory considerations: align with relevant regulatory guidelines; prepare comprehensive safety dossier; include mechanistic rationale for design choices; document risk mitigation strategies.
