# Supplementary figures and images for: Analyzing Molecular Determinants of Nanodrugs’ Cytotoxic Effects
Source: Int J Mol Sci. 2025 Jul 11;26(14):6687. doi: 10.3390/ijms26146687 (PMC12294593; doi:10.3390/ijms26146687)

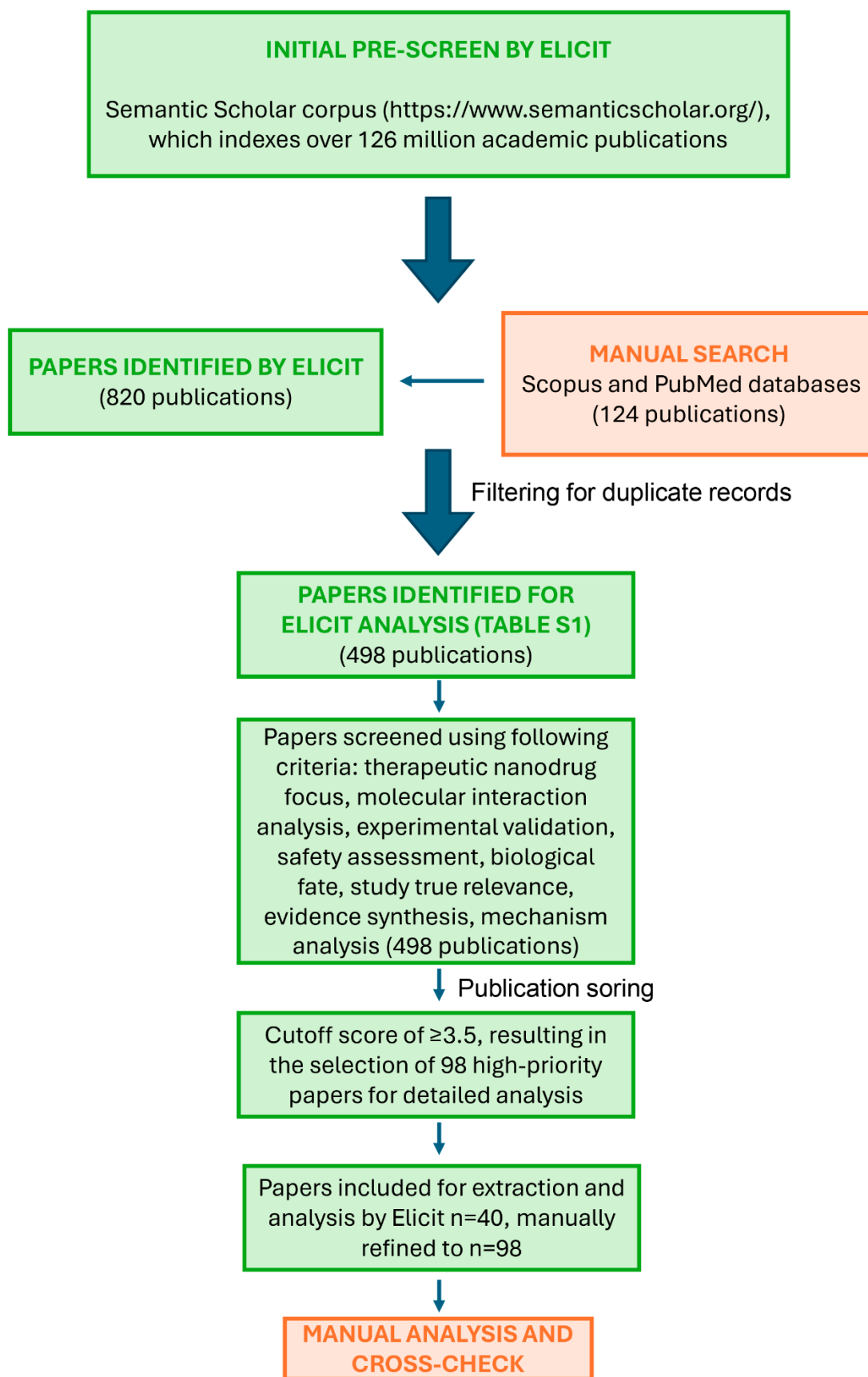

Supplementary Figure S1. Flowchart of publications selection, screening, and data extraction.

Supplement: Supplementary file 1 [file ijms-26-06687-s001.zip › Figure S1.pdf]
